# Causal role of immunophenotypes in HIV-1 acquisition: insights from Mendelian randomization analysis

**DOI:** 10.1038/s41598-025-07962-y

**Published:** 2025-07-02

**Authors:** Nan Li, Chuan He

**Affiliations:** 1 State Key Laboratory for Diagnosis and Treatment of Infectious Diseases, Shenyang, Liaoning China; 2 NHC Key Laboratory of AIDS Prevention and Treatment, Shenyang, Liaoning China; 3 National Clinical Research Center for Laboratory Medicine, Shenyang, Liaoning China; 4 Department of Laboratory Medicine, The First Hospital of China Medical University, 155 Nanjingbei Street, Heping District, Shenyang, 110001 Liaoning China

**Keywords:** HIV-1, Mendelian randomization, Peripheral blood, Genome-wide association study, Immunophenotypes, Immunotherapy, Infectious diseases, HIV infections

## Abstract

The acquisition of human immunodeficiency virus (HIV) is influenced by environmental and genetic factors, such as viral inoculum dose, host behavior, and immune responses. Despite advances in understanding HIV pathogenesis, no effective vaccine exists, underscoring the urgent need to deepen our comprehension of host immune mechanisms to enhance preventive strategies. Genetic predisposition and certain immunity characteristics of the host might play essential roles in the risk of HIV-1 acquisition. Mendelian randomization (MR) and colocalization analysis are utilized to investigate the causal relationships between immune responses and HIV-1 risk, aiming to identify targets for potential eradication strategies. We employed a two-sample MR approach to explore the causal links between 731 immunophenotypes and HIV-1 acquisition, using genetic variants from publicly available GWAS summary statistics as instrumental variables. Sources included GWAS data for immune traits and a meta-analysis from European cohorts for HIV-1 acquisition. We validated our findings using Summary-data-based MR analysis, integrating eQTL and mQTL data from the GTEx project. Bayesian colocalization analysis was conducted to identify shared causal variants. Functional and pathway enrichment analysis employing Metascape and Enrichr websets were performed to elucidate potential biological pathways linking immunephenotypes to HIV-1 risk. Our MR analysis identified significant causal associations between 26 specific immunophenotypes and HIV-1 acquisition, indicating a causal association with HIV risk. Colocalization analysis showed that none demonstrated genome-wide evidence of genetic colocalization (regional PP.H4.abf > 0.70). SMR and HEIDI analysis confirmed pleiotropic associations, particularly noting CCR2 on granulocytes as significant. Functional enrichment analysis of 39 SMR-identified genes revealed critical pathways linking immunophenotypes to HIV-1 susceptibility. The “NABA MATRISOME ASSOCIATED” canonical pathway emerged as the most significant pathway using Metascape. Protein–protein interaction networks demonstrated 5 functionally cohesive clusters. Multi-database analyses using Enrichr Analysis revealed functionally similar pathway enrichment: Cell cycle regulation (“G2 Phase”, Reactome; “G1/S Control”, WikiPathways), shedding light on crucial immune regulation mechanisms potentially instrumental in HIV-1 acquisition.

## Introduction

The human immunodeficiency virus (HIV), the causative agent of acquired immunodeficiency syndrome (AIDS), remains a significant global public health and socio-economic concern since its emergence in the early 1980s. The disease accounts for 1.5 million new infections and 1 million deaths globally each year^1^, and approximately 39 million people currently live with HIV/AIDS^2^. HIV-1 acquisition is a complex trait that depends on environmental and genetic factors, including the dose of viral inoculum^3^ and host behavioral, cellular, and immune parameters moderating susceptibility to infection, viral control, and systemic spread ^4^. Although there have been many advances in the development of successful prevention options (i.e., pre- and post-exposure prophylaxis (PrEP/PEP))^5^, an effective vaccine to prevent systemic infection remains elusive, underscoring the critical need for a deeper understanding of the mechanisms underlying host immunity to HIV-1.

Studies have shown that several risk factors, such as genetic predisposition, systemic inflammation, and cytokine dysregulation, might play essential roles in the risk of HIV-1 acquisition^6–8^. Recently, accumulating evidence has suggested that systemic immunity is related to the risk of HIV acquisition^9,10^. A prospective cohort study suggested that changes in imbalances of systemic and mucosal immunity increase HIV-1 acquisition risk^11^. In addition, the C–C motif chemokine receptor 5 (CCR5) has been unequivocally demonstrated to play an essential role in HIV-1 susceptibility, as evidenced by the discovery of the CCR5Δ32 mutation conferring resistance to infection^12,13^. The C–C motif chemokine receptor 2 (CCR2) allele is a prominent receptor for the Monocyte Chemoattractant Protein (MCP) group of C–C chemokines and is among the most important genetic factors known to be associated with host protection against HIV-1 infection^14^. The C-C motif chemokine ligand 17 (CCL17) is known to regulate the development and maturation of T-cells in the thymus, as well as their trafficking during inflammation^15^. A genome-wide association study including 13,581 individuals found that a higher genetic risk for HIV-1 acquisition was associated with lower levels of CCL17^4^. It was hypothesized that increased CCL17 levels may increase the inflow of inflammatory cells, which could help eliminate HIV-1 infected cells before the establishment of a systemic infection. In summary, despite recent advances in the understanding of AIDS virus pathogenesis, which immune cell subsets support the establishment of HIV-1 infection and replication in vivo remains incompletely understood. However, traditional observational studies cannot easily establish whether such associations are causal, due to issues like confounding and reverse causation.

Mendelian randomization (MR) is a genetic method that applies genetic variants associated with the exposure as instrumental variables (IVs) in non-experimental design to assess the causal effect of the exposure on the outcome^16^. Since genetic variants are presumed to be inherited randomly and alleles are not influenced by diseases, this method can minimize the influence of confounding factors and reverse causation bias^17^. Therefore, MR has been widely applied in identifying causal relationships between risk factors and diseases. In addition, colocalization is an essential analytical method for exploring the common causal molecular mechanism among different diseases and disease-related intermediate phenotypes^18^.

Through a comprehensive two-sample MR analysis utilizing data from multiple large cohorts, this study aims to systematically investigate the potential causal relationships between immunophenotypes and HIV-1 susceptibility. Our findings are expected to contribute to the identification of host mechanisms that modulate HIV-1 acquisition and potentially unveil novel strategies for combating and ultimately eradicating HIV-1.

## Materials and methods

### Study design

**Fig. 1:**
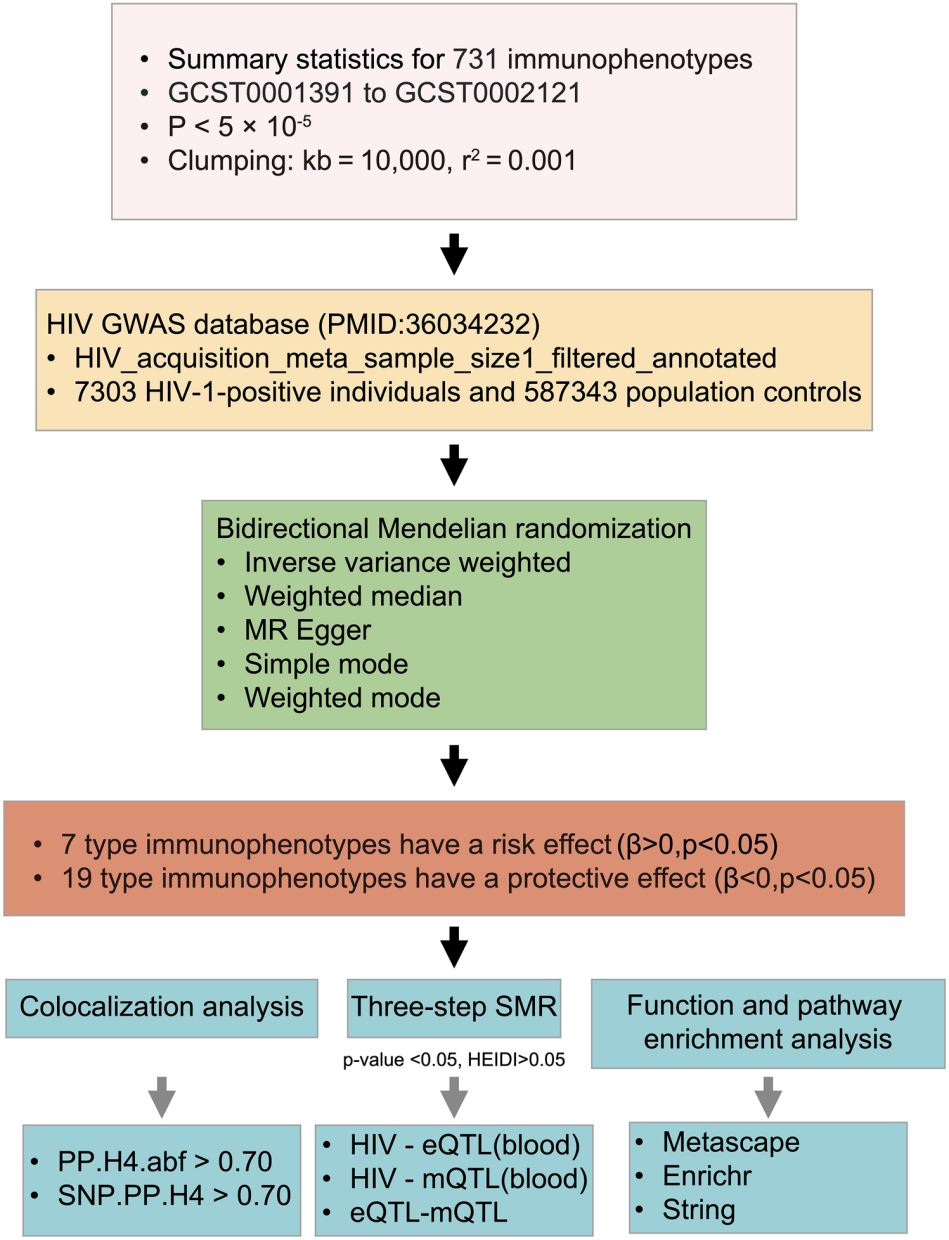
Flowchart of the study design.

We conducted a two-sample Mendelian Randomization (MR) analysis to evaluate the causal relationship between 731 immunophenotypes and HIV-1 acquisition. Genetic variants from publicly available GWAS summary statistics served as instrumental variables in this causal inference. The MR analysis relied on three core assumptions: the relevance of genetic variation to exposure, independence from confounders, and the exclusive effect of the genetic instruments on the outcome through exposure^19^. Since we used publicly available GWAS summary statistics, no additional ethical approval was required. Figure 1 shows the overall design of our study.

### Data sources for exposure and outcome

#### GWAS summary statistics of immunophenotypes

GWAS summary statistics for each immune trait are publicly available from the GWAS Catalog (accession numbers from GCST0001391 to GCST0002121)^20^. We standardized the immunophenotype GWAS summary statistics to include the following columns: Chromosome (chr_col), Base_pair_location (pos_col), Effect_allele (effect_allele_col), Other_allele (other_allele_col), N (samplesize_col): Sample size, Effect_allele_frequency (eaf_col), Beta (beta_col), Standard_error (se_col), P_value (pval_col), RSIDs (snp_col). A total of 731 immunophenotypes including absolute cell (AC) counts (n = 118), median fluorescence intensities (MFI) reflecting surface antigen levels (n = 389), morphological parameters (MP) (n = 32) and relative cell (RC) counts (n = 192) were included. Specifically, the MFI, AC and RC features contain B cells, CDCs, mature stages of T cells, monocytes, myeloid cells, TBNK (T cells, B cells, natural killer cells) and Treg panels, while the MP feature contains CDC and TBNK panels. The original GWAS on immune traits was performed using data from 3757 European individuals, and with no overlapping cohorts. Approximately 22 million single nucleotide polymorphisms (SNPs) genotyped with high-density arrays were imputed using Sardinian sequence-based reference panel^21^ and associations were tested after adjusting for covariates (i.e., sex, age).

#### GWAS summary statistics of HIV-1 acquisition

The GWAS summary data of HIV-1 acquisition were obtained from a meta-analysis of GWAS including 7303 HIV-1-positive individuals and 587,343 population controls of European populations^22^. We standardized the HIV GWAS summary statistics to include the following columns: CHR (snp_col), BP (pos_col), A1 (effect_allele_col), A2 (other_allele_col), P (pval_col), RSID (snp_col), MAF_dbsnp151: converted to match the effect allele frequency (eaf_col), BETA (beta_col), SE (se_col). Briefly, HIV-1 acquisition is defined as a binary phenotype, which corresponds to whether a person is currently diagnosed as HIV-1 positive. The genetic differences between HIV-1 positive and HIV-1 negative individuals were studied to determine the genetic variation related to HIV-1 acquisition, and these differences were defined according to immune reactivity or self-report. The data of meta-analysis includes FinnGen Public Data^23^ (data release 5, trait ID AB1_HIV, 357 cases and 218,435 population controls) and data from the UK Biobank (from the Neale lab, data release 3, trait ID 20002_1439 HIV/AIDS, 285 cases and 360,856 population controls)(http://www.nealelab.is/uk-biobank/). Additionally, the data include those Johnson et al. (327 HIV-1-positive cases and 805 HIV-1-negative controls)^24^and the data of McLaren et al. (6334 HIV-1-positive cases and 7247 population controls)^25^.

### Instrumental variable selection

We identified SNPs linked to outcomes and immunophenotypes using a significance threshold of *P* < 5 × 10^−5^, followed by clumping to mitigate linkage disequilibrium (kb = 10,000, r^2^ = 0.001)^26,27^. Each SNP’s strength was evaluated using the F-statistic, with SNPs boasting an F-statistic > 10 deemed sufficiently strong as IVs^28^. Additionally, using these SNPs, reverse MR analysis was performed to investigate the effect of genetic predisposition to HIV-1 acquisition on immunophenotypes levels.

### Bidirectional Mendelian randomization

We used the inverse-variance weighted (P-IVW < 0.05) method as the primary analysis to evaluate the causal link between 731 immunophenotypes and HIV-1 acquisition by combining the β values and the standard errors of the causal estimates^29^. Cochran’s Q statistic and corresponding *P* values were used to test the heterogeneity among selected IVs. If the null hypothesis was rejected, the random effects IVW method was used instead of the fixed-effects IVW method^30^. To assess the robustness of the primary analyses, we applied several sensitivity analyses. First, we used the simple-median method and weighted-median method to estimate the potential causal effects when IVs violated standard assumptions^31^. Furthermore, MR-Egger regression was performed to assess the presence of directional pleiotropy, with *P*-values for intercept < 0.05 considered statistically significant and indicating the presence of horizontal pleiotropy^32^. Leaveone-out (LOO) sensitivity analysis was then used to determine the association of individual SNPs and whether the results were driven by any single SNP^31^. The analyses were carried out using the TwoSampleMR package (version 0.5.6) implemented in R (version 3.4). The forest plots were drawn using the Forestplot package (version 2.0.1). Statistical significance was defined as a *P* value < 0.05^33^.

### Bayesian colocalization analysis

To assess whether two associated signals (immunophenotypes and HIV-1 acquisition) were consistent with a shared causal variant, thereby distinguishing the confounding of linkage disequilibrium, we employed summary statistics of immunophenotype and HIV-1 acquisition meta-GWASs to perform Bayesian colocalization analysis using the “coloc” package^34^ and the LocusCompareR package. To prepare data for Bayesian colocalization analysis, we implemented the following preprocessing steps: Removal of duplicate RSID (snp_col) to prevent redundancy. Exclusion of rows lacking SNP information to maintain data integrity. Parsing of rows containing multiple SNPs separated by commas to ensure each SNP is represented individually.Addition of the sample size column (samplesize_col) from the GWAS data. Saving the processed dataset in a tab-delimited text (.txt) format for compatibility with colocalization analysis package. The colocalization analysis included five hypotheses: (i) There was no causal variant for either immunophenotype or HIV-1 acquisition in the genomic locus (H0); (ii) There was one causal variant for immunophenotype (H1); (iii) There was one causal variant for HIV-1 acquisition only (H2); (iv) There were two distinct causal variants for immunophenotype and HIV-1 acquisition (H3); (v) There was a shared causal variant for immunophenotype and HIV-1 acquisition (H4). The prior probability was set as 1 × 10^−6^ that a random variant is causal to both GWAS and defining colocalization. The posterior probability for H4 (PP4) that was higher than 70% under different priors and windows was considered strong evidence of colocalization^35^.

### Summary-data-based MR (SMR) Analysis

SMR analysis was further conducted as a complementary method to verify the causal associations between immunophenotypes and HIV-1 acquisition^36^, integrating both expression quantitative trait loci (eQTL) and methylation quantitative trait loci (mQTL) data. Data preprocessing followed the methodology used in our Bayesian colocalization analysis of HIV GWAS data. Using SMR software (smr_Mac_v1.03) with default parameters, we defined a 2 Mb window centered on each probe for cis-eQTL/mQTL selection and applied Bonferroni correction to establish significance thresholds (*Adjusted-P-value* < 0.05 for SMR) for multiple testing. The heterogeneity in dependent instruments (HEIDI) test (*P-value* > 0.05) distinguished pleiotropy-driven associations from those confounded by linkage disequilibrium, excluding SNPs in strong linkage disequilibrium with the top-associated QTL. This approach ensured that observed immunophenotype–HIV-1 associations were attributable to shared causal variants rather than genetic linkage.

#### eQTL data source

We utilized the V8 release of the Genotype-Tissue Expression (GTEx) project's eQTL summary data^37^. This dataset encompasses cis-eQTL summary statistics across 49 human tissues, focusing on single nucleotide polymorphisms (SNPs) located within 1 megabase (Mb) of the transcription start sites. For our analysis, we employed the GTEx_V8_cis_eqtl_summary_lite (506 MB) dataset in SMR binary (BESD) format.

#### mQTL data source

Our analysis also incorporated mQTL summary data from McRae et al., spanning two cohorts: the Brisbane Systems Genetics Study (BSGS) and the Lothian Birth Cohorts (LBC), with a combined sample size of 1980 individuals of European descent^36,38^. DNA methylation levels were assessed using Illumina HumanMethylation450 BeadChips. The LBC_BSGS_meta_lite dataset, a meta-analysis of BSGS and LBC cohort data in SMR binary (BESD) format (241 MB), was used. This dataset focuses on DNA methylation probes associated with at least one cis-mQTL (*P* < 5 × 10^–8^) and includes only SNPs within a 2 Mb range from each probe, ensuring a targeted yet comprehensive examination of methylation changes associated with genetic variants.

These integrated eQTL and mQTL datasets facilitated a nuanced exploration of the genetic determinants of gene expression and DNA methylation, respectively, allowing for a robust analysis of potential causal relationships between genetic variants and HIV-related phenotypes through the SMR approach.

### Functional and pathway enrichment analysis

To elucidate potential mechanisms underlying immunophenotype-mediated HIV-1 susceptibility, we performed functional and pathway enrichment analyses on genes identified through SMR analysis.

1. Pathway and process enrichment analyses were conducted using Metascape^39^(https://metascape.org). Gene ontology (GO) and pathway analyses were performed using multiple ontology sources, including KEGG Pathways^40^, GO Biological Processes, Reactome Gene Sets, Canonical Pathways, CORUM, WikiPathways, and PANTHER Pathway.

All genes within the genome served as the enrichment background. Terms meeting the criteria (*P*-value < 0.01, a minimum gene count of 3, and an enrichment factor > 1.5) were considered significantly enriched. Statistical significance was calculated using cumulative hypergeometric distribution.

The enriched terms were hierarchically clustered using Kappa scores (> 0.3 similarity). The most statistically significant term within each cluster represented that cluster.

(2) Protein–Protein Interaction (PPI) Analysis.

Protein–protein interaction analysis was conducted using the STRING database (https://cn.string-db.org/). Interaction pairs were identified based on known and predicted interactions. Key interaction nodes and pairs were recorded for further biological interpretation.

(3) Enrichr Analysis.

Scatter plot visualizations and bar charts of enriched terms from the Reactome_Pathways_2024 and WikiPathways_2024_Human gene set libraries were performed using Enrichr^41–43^ (https://maayanlab.cloud/Enrichr). Term frequency-inverse document frequency (TF-IDF) values were calculated, followed by dimension reduction with Uniform Manifold Approximation and Projection (UMAP). Clusters were identified using the Leiden algorithm. Statistical significance of enrichment was reported as -log10 (*P*-values).

## Results

### Causal effect of immunophenotypes on HIV-1 acquisition

To investigate the causal association between immune characteristics and HIV-1 infection, we performed two-sample MR analyses using the IVW method as the primary screening approach. For four exposure immunophenotypes, CM DN (CD4-CD8-) AC[GCST90001563], CD28 on CD28 + DN (CD4-CD8-)[ GCST90001895], CD25 on CD28 + CD4 + [GCST90001962], and CD45RA on CD39 + resting Treg[GCST90002103], only one genome-wide significant SNP (*P* < 5 × 10^−5^) was available as a valid IV. These immunophenotypes were excluded from MR analyses because single-SNP estimates are highly susceptible to bias from undetected horizontal pleiotropy, sensitivity analyses require at least two IVs and were not feasible. In total, we calculated the causal effects of 727 immunophenotypes on HIV (Supplementary Table 1). Our MR analysis revealed a significant causal association between 26 immunophenotypes and acquiring HIV-1 (*P* < 0.05). These associations spanned various cell groups: seven in B cells, two in conventional dendritic cells (cDC), three in mature T cells, five in myeloid cells, five in TBNK cells, and four in regulatory T cells (Tregs) (Supplementary Fig. 1).

**Fig. 2:**
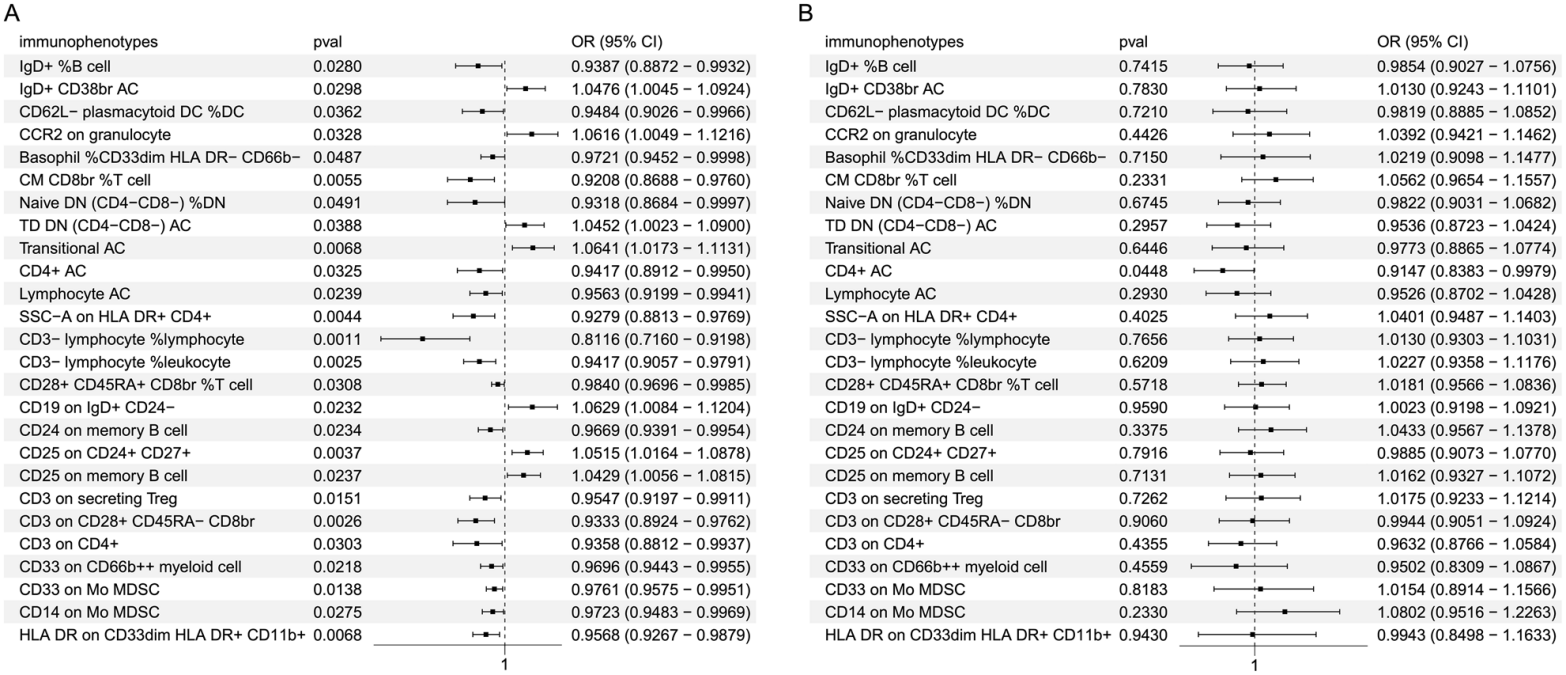
Estimation of the causal relationship between immunophenotypes and HIV. Forest plots were employed to depict results of the MR investigation, showing the causal effects of genetically predicted protective and risk immunophenotypes on HIV using the IVW method. (**A**) Forward MR analysis, where immunophenotypes were considered as exposures and HIV as the outcome. (**B**) Reverse MR analysis: where HIV was considered as the exposure and immunophenotypes as the outcome.

Specifically, among these 26 immunophenotypes, the increase in 19 kinds of immunophenotypes (IgD + %B cell, CD24 on memory B cell, CD62L- plasmacytoid DC %DC, CM CD8br %T cell, Naive DN (CD4-CD8-) %DN, Basophil %CD33dim HLA DR- CD66b-, CD33 on CD66b +  + myeloid cell, CD33 on Mo MDSC, CD14 on Mo MDSC, HLA DR on CD33dim HLA DR + CD11b + , CD4 + AC, Lymphocyte AC, CD3- lymphocyte % lymphocyte, CD3- lymphocyte %leukocyte, SSC-A on HLA DR + CD4 + , CD28 + CD45RA + CD8br %T cell, CD3 on secreting Treg, CD3 on CD28 + CD45RA- CD8br and CD3 on CD4 + Treg) is negatively correlated with HIV-1 risk(OR < 1), indicating that the higher levels of thess immunophenotypes are associated with the lower HIV-1 susceptibility (Fig. 2A). However, the increase of seven kinds of immunophenotypes (IgD + CD38br AC, Transitional AC, CD19 on IgD + CD24-, CD25 on CD24 + CD27 + , CD25 on memory B cell, CCR2 on granulocyte, TD DN (CD4-CD8-) AC) is associated with increased susceptibility to HIV-1(OR > 1) (Fig. 2A). These associations were consistent across other analyses, including the weighted mode, weighted median and MR-Egger.

Our heterogeneity and pleiotropy analyses showed that, except for rs76604940 in GCST90001688 (Relative count, Treg CD28 + CD45RA + CD8br %T cell), the results were overall robust, with no single SNP having a disproportionate influence on the outcomes (heterogeneity *P* > 0.05, pleiotropy *P* > 0.05) (Table 1, Supplementary Figs. 2 and 3). To further excluding the possibility of reverse causality, we performed reverse MR on the 26 immunophenotypes, and found that no statistical significance except CD4 AC cells (*P* < 0.05) (Fig. 2B).

**Table 1:** The heterogeneity and pleiotropic analyses for causality between immunophenotypes and HIV.

	Heterogeneity test				Pleiotropic test		
Exposures	Methods	Q	Q_dif	Pval	Methods	SE	Pval
IgD + %B cell	MR.Egger	0.643929637	3	0.886306075	Egger_Intercept	0.011695481	0.496969894
	IVW	1.238052584	4	0.871794648			
IgD + CD38br AC	MR.Egger	9.390441725	12	0.669269114	Egger_Intercept	0.0092191	0.512747224
	IVW	9.845461276	13	0.706520088			
CD62L- plasmacytoid DC %DC	MR.Egger	11.57713736	9	0.238211098	Egger_Intercept	0.01250454	0.804180118
	IVW	11.6610229	10	0.308383311			
Basophil %CD33dim HLA DR- CD66b-	MR.Egger	12.06593318	11	0.358707702	Egger_Intercept	0.008125787	0.834090958
	IVW	12.1163963	12	0.436377586			
CM CD8br %T cell	MR.Egger	8.024716603	8	0.431059456	Egger_Intercept	0.025086421	0.336278587
	IVW	9.074318188	9	0.430442463			
Naive DN (CD4-CD8-) %DN	MR.Egger	11.35721505	9	0.252015958	Egger_Intercept	0.018942989	0.301536002
	IVW	12.87306123	10	0.230855978			
TD DN (CD4-CD8-) AC	MR.Egger	8.775265155	12	0.721996475	Egger_Intercept	0.011563367	0.827104745
	IVW	8.825100651	13	0.786020845			
Transitional AC	MR.Egger	11.47175848	13	0.571360176	Egger_Intercept	0.008545089	0.240825991
	IVW	12.98239262	14	0.527910521			
CD4 + AC	MR.Egger	6.590300845	9	0.679689528	Egger_Intercept	0.022400606	0.233738423
	IVW	8.219700192	10	0.607386512			
Lymphocyte AC	MR.Egger	2.228950146	6	0.897484028	Egger_Intercept	0.008123094	0.612198343
	IVW	2.514662468	7	0.925988925			
CD3- lymphocyte %leukocyte	MR.Egger	7.943596037	7	0.337593844	Egger_Intercept	0.007698984	0.392015129
	IVW	8.887789637	8	0.351850222			
CD28 + CD45RA + CD8br %T cell	MR.Egger	8.367066768	10	0.593029744	Egger_Intercept	0.004690224	0.593690482
	IVW	8.670732841	11	0.652255623			
CD19 on IgD + CD24-	MR.Egger	12.8578492	10	0.231728605	Egger_Intercept	0.014583887	0.758580726
	IVW	12.98615281	11	0.29423142			
CD24 on memory B cell	MR.Egger	5.581227014	11	0.899795627	Egger_Intercept	0.005767217	0.068767389
	IVW	9.649589448	12	0.646671474			
CD25 on CD24 + CD27 +	MR.Egger	8.840961748	14	0.841101597	Egger_Intercept	0.008413337	0.953104271
	IVW	8.84454634	15	0.885504276			
CD25 on memory B cell	MR.Egger	10.30211171	11	0.503434238	Egger_Intercept	0.00834607	0.376833363
	IVW	11.15025648	12	0.516087507			
CD3 on secreting Treg	MR.Egger	4.056637667	6	0.66901185	Egger_Intercept	0.00828587	0.663667495
	IVW	4.265597427	7	0.748727904			
CD3 on CD28 + CD45RA- CD8br	MR.Egger	6.612784709	7	0.470277511	Egger_Intercept	0.011963019	0.372685924
	IVW	7.519561176	8	0.481747727			
CD3 on CD4 +	MR.Egger	12.73657245	10	0.238775559	Egger_Intercept	0.015319083	0.557511395
	IVW	13.20553327	11	0.280104449			
CD33 on CD66b + + myeloid cell	MR.Egger	5.258262284	5	0.385181347	Egger_Intercept	0.010236541	0.134463916
	IVW	8.606097233	6	0.19697258			
CD33 on Mo MDSC	MR.Egger	5.002129974	8	0.757348454	Egger_Intercept	0.00622216	0.176424425
	IVW	7.200705271	9	0.616231896			
CCR2 on granulocyte	MR.Egger	2.926201174	5	0.711362421	Egger_Intercept	0.015755406	0.939958319
	IVW	2.932471155	6	0.817273472			
CD14 on Mo MDSC	MR.Egger	7.275235473	12	0.838901726	Egger_Intercept	0.010545632	0.985732624
	IVW	7.27556885	13	0.887397329			
SSC-A on HLA DR + CD4 +	MR.Egger	6.896473534	7	0.439738113	Egger_Intercept	0.015119111	0.992350678
	IVW	6.89657223	8	0.547831861			
HLA DR on CD33dim HLA DR + CD11b +	MR.Egger	2.460324162	6	0.87288133	Egger_Intercept	0.011624914	0.826879922
	IVW	2.512515685	7	0.926151676			
CD3- lymphocyte %lymphocyte	MR.Egger	NA	NA	NA	Egger_Intercept	NA	NA
	IVW	NA	NA	NA			

### Colocalization analysis

Among the 26 immunophenotypes showing potential causal relationships with HIV in our Mendelian randomization (MR) analysis, none demonstrated genome-wide evidence of genetic colocalization (regional PP.H4.abf > 0.70). However, at the SNP level, we identified four immunophenotypes with strong evidence of shared causal variants (SNP.PP.H4 > 0.70): (1) CD33 on Mo MDSC, (2) CD25 on memory B cell, (3) TD DN (CD4-CD8-) AC, and (4) CCR2 on granulocyte (Supplementary Fig. 4.Notably, rs445 (CCR2 on granulocytes) showed the highest SNP-level posterior probability (SNP.PP.H4 = 0.9999). Importantly, for CD33 on Mo-MDSCs, the lead variant rs3865444 is located within the CD33 gene locus itself. In contrast, the other three immunophenotypes showed no obvious shared biological pathways between their mapping genes: CCR2's rs445 maps to CDK6, TD DN's rs6729180 maps to GALM, and CD25's rs1887027 shows no clear functional connection. These findings underscore the need for further functional validation of these candidate SNPs.

### SMR analysis

Given that genetic effects on HIV susceptibility might be mediated by eQTLs and mQTLs(which modulate DNA methylation patterns), we applied SMR to investigate pleiotropic associations between these molecular traits and HIV-1 acquisition.

**Fig. 3:**
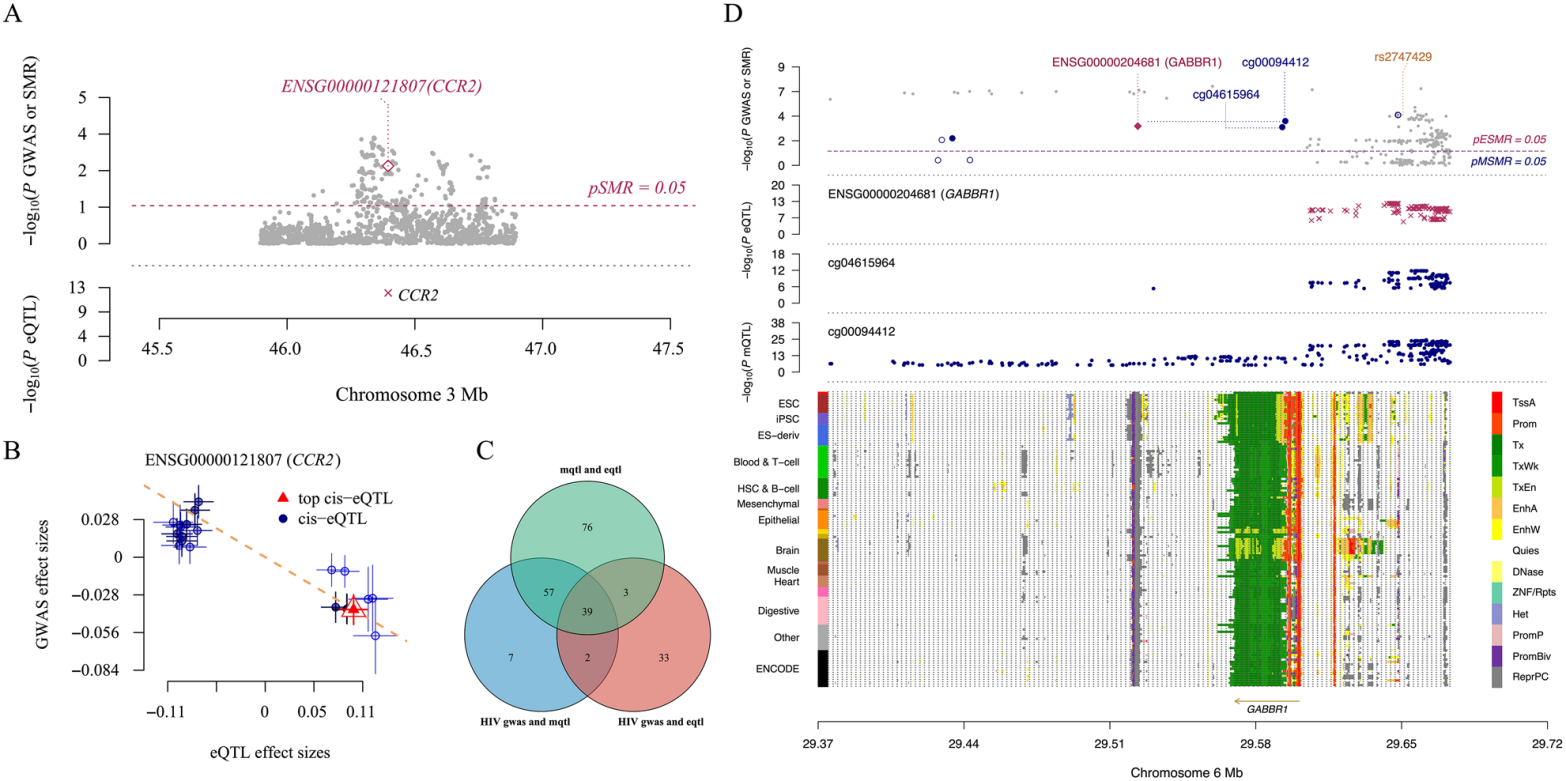
Analysis of Pleiotropic Associations between Genetic Variants and HIV-1 Acquisition Using SMR & HEIDI Methods. (**A** and **B**) Association between CCR2 gene expression and HIV-1 acquisition (*P* < 0.05). (**C**) Venn diagram analysis of three distinct SMR analyses based on identical SNP and gene information. (**D**) Multi-omics analyses of GABBR1 (rs2747429) and HIV.

In our eQTL-HIV SMR analysis, we identified CCR2 on granulocytes as significantly associated with HIV-1 acquisition (pSMR < 0.05, Fig. 3A and B).To further disentangle causal mechanisms, we integrated two complementary SMR analyses: mQTL-HIV SMR (methylation-to-HIV) and eQTL-mQTL SMR (expression-to-methylation). By intersecting results from all three approaches (Fig. 3C), we identified 39 loci where genetic instruments and their corresponding genes (cis-eQTL/cis-mQTL) consistently pointed to causal relationships.

To rank these 39 loci, we defined the top gene-SNP pair as the locus with the smallest sum of eQTL and mQTL p-values (i.e., peQTL + pmQTL).This approach prioritizes variants showing strong concordant effects across both expression and methylation levels, thereby enhancing confidence in their biological relevance, and reducing the likelihood of spurious associations driven by a single dataset. The top-ranked locus was GABBR1 : sum-p = 3.21E-04, tagged by rs2747429 (Fig. 3D). Notably, CCR2 (sum-p = 2.25E-03) and its lead SNP rs6441957 ranked second (Table 2), reinforcing its role in HIV susceptibility.

**Table 2:** SMR analysis of eQTL, mQTL, and HIV.

Gene, SNP	eQTL p	mQTL p	sum of eQTL and mQTL p value
GABBR1, rs2747429	2.59E-04	6.26E-05	3.21E-04
CCR2, rs6441957	2.19E-03	5.95E-05	2.25E-03
CCND2, rs3217860	1.79E-03	1.02E-03	2.81E-03
SULF2, rs78055051	2.62E-03	2.21E-03	4.83E-03
ZYX, rs2242601	2.49E-03	2.37E-03	4.85E-03
GAA, rs12450199	5.84E-03	7.45E-03	1.33E-02
FES, rs4932373	9.81E-03	8.00E-03	1.78E-02
PNKP, rs2257103	9.35E-03	1.04E-02	1.97E-02
MDGA1, rs9349050	8.13E-03	1.20E-02	2.01E-02
MX1, rs459498	9.84E-03	1.33E-02	2.31E-02
MMP25, rs10431961	9.76E-03	1.37E-02	2.35E-02
NECAP2, rs12031735	1.38E-02	1.27E-02	2.65E-02
HMGN4, rs9467773	1.52E-02	1.46E-02	2.98E-02
AMH, rs4807216	2.20E-02	2.04E-02	4.24E-02
PRDX2, rs3786712	2.17E-02	2.10E-02	4.27E-02
FLYWCH1, rs9928222	1.93E-02	2.41E-02	4.34E-02
BMP8A, rs79473113	2.84E-02	2.55E-02	5.40E-02
ZNF514, rs11686534	2.30E-02	3.15E-02	5.46E-02
IGFBP2, rs16856445	2.40E-02	3.09E-02	5.49E-02
PLEKHG3, rs1998120	2.75E-02	2.84E-02	5.59E-02
C17orf97, rs7502594	2.51E-02	3.09E-02	5.60E-02
RBMS2, rs3782235	2.85E-02	2.82E-02	5.67E-02
OLFM4, rs17552047	2.74E-02	3.13E-02	5.87E-02
TMEM127, rs2301707	3.59E-02	2.79E-02	6.38E-02
IL15RA, rs8177637	2.86E-02	3.81E-02	6.67E-02
EPHB2, rs2043970	3.24E-02	3.53E-02	6.77E-02
S100A9, rs3014874	3.53E-02	3.51E-02	7.04E-02
GTF3A, rs1049302	4.12E-02	3.53E-02	7.65E-02
C1orf109, rs11264091	4.06E-02	3.69E-02	7.75E-02
RIN1, rs7950463	4.07E-02	3.99E-02	8.06E-02
ZNF155, rs62116613	4.11E-02	4.03E-02	8.14E-02
BTBD11, rs1991310	4.62E-02	3.76E-02	8.38E-02
ACCS, rs2074038	4.23E-02	4.27E-02	8.50E-02
DENND2C, rs114829951	4.50E-02	4.37E-02	8.87E-02
ANKLE1, rs35006574	4.91E-02	4.28E-02	9.18E-02
CCNA1, rs4941839	4.56E-02	4.66E-02	9.23E-02
DECR2, rs1204503	4.66E-02	4.72E-02	9.37E-02
LGALS12, rs1404504	4.94E-02	4.71E-02	9.65E-02
ANXA6, rs11750621	4.98E-02	4.78E-02	9.77E-02

### Functional and pathway enrichment analysis

**Fig. 4:**
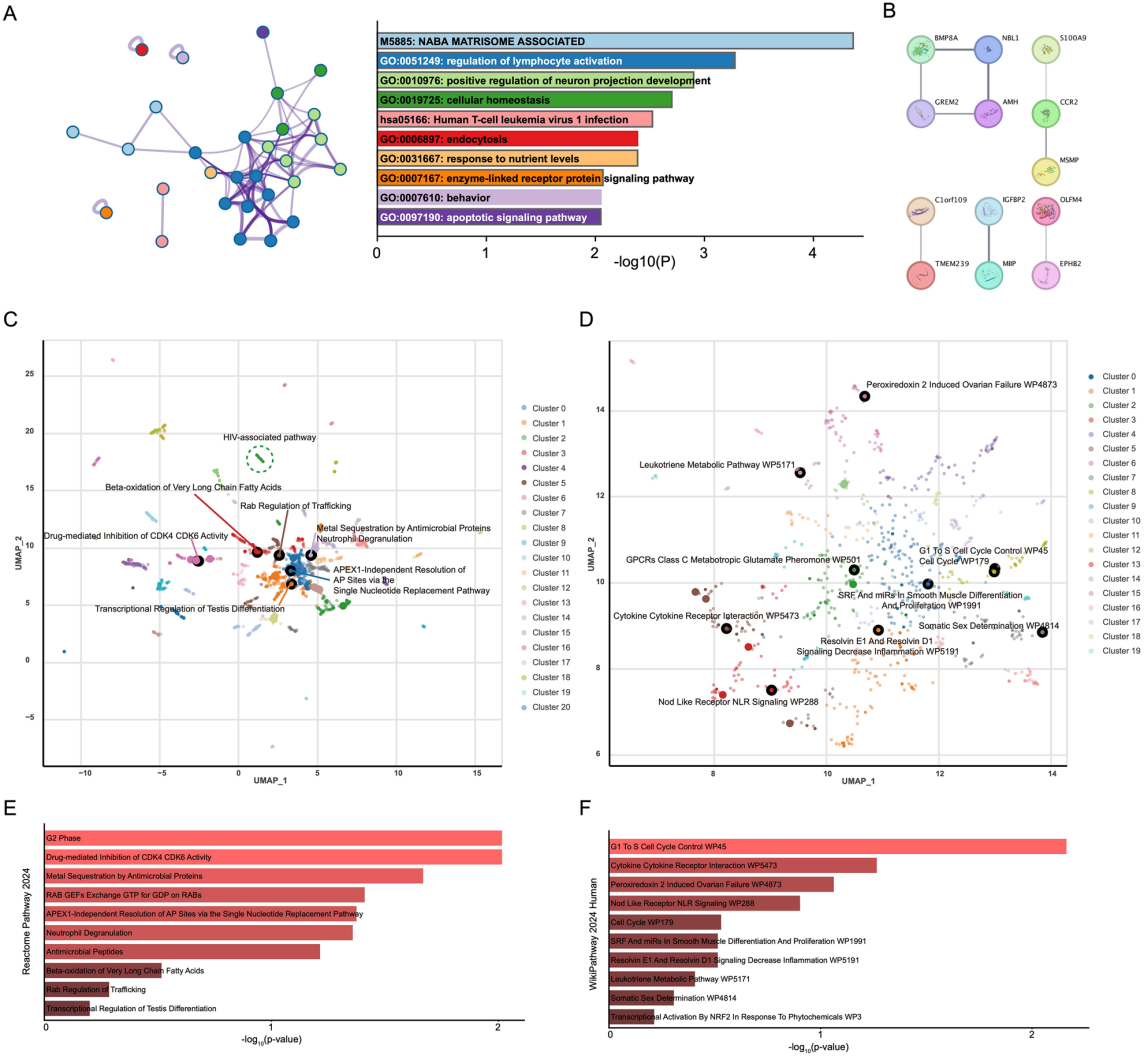
Functional Enrichment and Network Analysis Highlighting Key Pathways and Biological Processes in HIV-1 Acquisition. (**A**) Pathway and process enrichment analysis performed using Metascape. The bar chart displays the top enriched terms. (**B**) PPI network constructed using the STRING database. Nodes represent proteins, and edges represent interactions based on STRING database analysis. (**C** and **D**) Scatter plots of enriched terms from the Reactome_Pathways_2024 (**C**) and WikiPathways_2024_Human (**D**) databases using Enrichr analysis. Each point represents an enriched term plotted based on UMAP dimensions. Clusters identified are indicated by color. Larger and darker points represent significantly enriched terms. (**E** and **F**) Bar charts showing the top 10 enriched terms from the Reactome_Pathways_2024 (**E**) and WikiPathways_2024_Human (**F**) databases, ranked by enrichment significance (-log10(*P*-value)).

We identified the top 10 enriched pathways and processes based on the provided list of 39 SMR genes using Metascape. The most significant term identified was the "NABA MATRISOME ASSOCIATED" canonical pathway (*P*-value = 10^−4.36^), which included 7 genes (17.95% of input genes). Other significantly enriched GO Biological Processes included regulation of lymphocyte activation, positive regulation of neuron projection development, cellular homeostasis, endocytosis, and apoptotic signaling pathway. A relevant KEGG pathway, "Human T-cell leukemia virus 1 infection," was also significantly enriched (Fig. 4A, Supplementary Table 2).The PPI analysis revealed 5 significant interactions among identified proteins, such as interactions between AMH and GREM2, BMP8A with NBL1, and CCR2 interactions with S100A9 and MSMP (Fig. 4B). These interactions suggest potential functional associations relevant to the biological processes identified in the enrichment analysis.

Scatter plot visualizations demonstrated clusters of related terms within Reactome (Fig. 4C) and WikiPathways (Fig. 4D) databases, highlighting biologically relevant groupings. Significantly enriched terms from Reactome included pathways such as"G2 Phase", and "Drug-mediated Inhibition of CDK4/CDK6 Activity"(Fig. 4E, Supplementary Table 3). WikiPathways analysis identified significant terms such as "G1 to S Cell Cycle Control," "Cytokine Cytokine Receptor Interaction," and "Nod Like Receptor (NLR) Signaling" (Fig. 4F, Supplementary Table 4).

## Discussion

Exposure to infectious agents does not always lead to a systemic infection. For instance, epidemiological studies prior to antiretroviral therapy indicated that up to two-thirds of individuals exposed to HIV-1 do not become infected^44,45^. Although the dose of viral inoculum^46^ and route^47^ of exposure are strong predictors of systemic infection, it has been hypothesized that host immune level differences also moderate susceptibility to viral entry, replication, and a systemic spread^4,48^. However, aside from CCR5, the host genetic factors involved in susceptibility to HIV-1 acquisition, particularly those related to common genetic variants, remain elusive.

Here, based on large publicly available genetic data, we explored causal associations between 731 immunophenotypes and HIV-1 acquisition. To our knowledge, this is the first two-sample MR analysis to explore the causal relationship between multiple immunophenotypes and HIV-1 acquisition. In this study, among four types of immunophenotypes (MFI, RC, AC, and MP), HIV-1 acquisition was found to have causal effects on 26 immunophenotypes. Among the 26 immunophenotypes, none demonstrated genome-wide evidence of genetic colocalization (regional PP.H4.abf > 0.70). However, at the SNP level, we identified four immunophenotypes with strong evidence of shared causal variants (SNP.PP.H4 > 0.70).

Our study found that the acquisition of HIV-1 increased with an increase in CCR2 on granulocyte. CCR2 has been shown to be one of the co-receptors of HIV found on the surface of the target cell and studied as genetic factors known to be associated with HIV infection^49^. A recent study indicated that children carrying the CCR2 64I allele were more likely to be HIV-infected than those carrying the wild type allele (CCR2 64V), with increased risk when the mothers also carried the mutation^50^. However, the association of CCR2 polymorphism with HIV transmission or disease progression remains highly controversial. A study carried out in a Guangxi Province population, a Chinese ethnic group, showed no significant difference in frequencies of CCR2-64I (χ2 = 1.795,* P* = 0.180) between the group of healthy individuals and the group of HIV-positive patients^51^. Ding et al. aiming to clarify the relationship between the polymorphism of CCR2-V64I and the risk of HIV-1 infection also found that it has no effect on susceptibility in the total population^49^. Scientists believe that the roles of CCR2 may differ in different populations due to variation in genetic backgrounds. This also implies that HIV acquisition or protection may be an association of several factors. Our results from large GWAS cohorts showed that an increase of CCR2 on granulocyte is related to increased susceptibility to HIV-1. Further study on its functional characteristics when the virus establishes infection will provide clues for the protective prevention of HIV-1.

TD DN (CD4-CD8-) AC, defined as CD3 T cells that lack CD4 and CD8 expression, derive either from the thymus by the escape of negative selection or from CD4^+^ T cells or CD8^+^ T cells in the periphery in response to antigenic stimulation^52–54^. DNT cells only account for a low frequency (1–and contain more HIV DN5%) of peripheral T cells in the general population, while increased frequency of peripheral DNT cells have been observed in autoimmune diseases, neoplastic diseases and infectious diseases^54–57^. Previous studies found that in AIDS patients, the frequency of DNT cells in the periphery increased significantly, being twice that of healthy individuals^55^. Both peripheral and pulmonary mucosal DNT cells have been reported to be latent HIV virus reservoirs^58^, however, whether HIV enters and infects DNT cells via HIV co-receptors remains unclear. Our research shows that there is a causal relationship between this immunophenotypes and HIV-1 susceptibility, which further supplements the understanding of their interaction. However, more cohort studies are still needed to verify this conclusion.

CD25( +) FoxP3( +) memory CD4 T cells, often referred to as regulatory CD4 T cells, are significantly more proliferative, and contain more HIV DNA than CD25(−) FoxP3(−) memory CD4 T cell subsets. The specific cellular characteristics of CD25( +) FoxP3( +) memory CD4( +) T cells probably facilitate efficient HIV infection in vivo and passage of HIV DNA to cell progeny in the absence of active viral replication^59^. While a causal association between increased CD25 on memory B cell instead of memory CD4 T cells and increased HIV-1 susceptibility was found in our study, the mechanism of its action with the virus is currently unknown. However, several studies indicated that although many of the HIV-associated defects improve with antiretroviral therapy (ART), excess immune activation and antigen-specific B cell function are still impaired in virologically controlled HIV-infected persons on ART, suggestting an effect on the germinal center reaction^60,61^. In addition, regarding the populations of memory B cells that circulate in the peripheral blood of elite controllers, that were demonstrated that more closely resemble those of healthy donors than those of HIV-infected individuals whose viremia is suppressed by ART^62^. Furthermore, an HIV-specific response enriched within HIV-infected individuals’ resting memory B cells, was observerd, with significantly higher frequencies in the elite controllers, despite a lower cellular HIV burden than in the ART group^63^. These findings confirm that extend observations that an intact humoral immune capacity may be responsible for a superior HIV-specific B-cell response. Our study found that the acquisition of HIV-1 increased with an increase in CD25 on memory B cell, and suggesting that a complex model for HIV-1 acquisition is moderated. More future mechanistic studies are still needed to clarify how CD25 on memory B cell promotes HIV infection.

Our results suggest that a higher level of CD33 on Mo MDSC was significantly associated with a lower risk of HIV-1 acquisition. Myeloid-derived suppressor cells (MDSCs) are a heterogeneous population of immature myeloid cells at various stages of differentiation (immature macrophages, granulocytes, dendritic cells, and other myeloid progenitors) that expand because of aberrant and sustained myelopoiesis under pathogenic conditions, such as cancer and inflammatory or infectious diseases^64–66^. In humans, the phenotype of MDSCs represents a population of cells with CD11b^+^CD33^+^ HLA-DR^−^, which are further divided into monocytic MDSCs (M-MDSCs) and granulocytic MDSCs (G-MDSCs) based on the differential expression of surface markers CD14 and CD15^65,67^. MDSCs suppress immune responses by the production of inflammatory and immunosuppressive molecules, including arginase 1 (ARG1), inducible nitric oxide synthase (iNOS), reactive oxygen species (ROS), and signal transducer and activator of transcription 3 (STAT3), all of which are important mediators of innate immune responses against pathogenic infections^68–70^. Previous studies reported that MDSCs expansion can inhibit T cell function in multiple disease models, including chronic HCV and HIV infections^71–74^. MDSCs expand and accumulate in people living with HIV, and this expansion correlates with disease progression^75,76^. However, the mechanisms that drive MDSCs differentiation and suppressive functions during the establishment of virus infection remain unclear. It is worth mentioning that studies have reported that the amplification of MDSC and the expression of immunosuppressive mediators are different between healthy people and HIV-infected people^77–79^. In healthy CD33 myeloid cells, their differentiation into MDSCs with immunosuppressive function was promoted due to the overexpression of HOXA transcript antisense RNA myeloid-specific 1 (HOTAIRM1) or HOXA1 gene, while silencing their expression in CD33 myeloid cells derived from people living with HIV weakens the differentiation and immunosuppressive function of MDSC^77,78^. Similarly, silencing RUNX1 overlapping RNA (RUNXOR) or runt-related transcription factor 1 (RUNX1) expression in MDSCs from people living with HIV attenuated MDSC expansion and immunosuppressive mediator expressions, whereas overexpressing RUNXOR in CD33 myeloid precursors from healthy subjects promoted their differentiation into MDSCs and enhanced the expression of suppressive mediators^79^. Our research found that the increase of CD33 on Mo MDSC was related to the decrease of HIV-1 acquisition [odds ratio (OR): 0.976, 95% confidence interval (CI): 0.958–0.995], and the above researches also confirmed our view. Therefore, further exploring the role and mechanism of CD33 on Mo MDSC in the establishment of HIV-1 infection may be beneficial to the prevention and control of HIV-1.

This study conducted a two-sample MR analysis based on the published results of large GWAS cohorts, with a large sample size of approximately 600,000 people, so it has high statistical efficiency. The conclusions of this study are based on genetic instrumental variables, and causal inference is made using a variety of MR analysis methods. The results are robust and were not confounded by horizontal pleiotropy and other factors. There are limitations to our study that should be acknowledged. First, even when multiple sensitivity analyses are performed, horizontal pleiotropy cannot be fully assessed. Second, due to the lack of individual information, we cannot conduct further stratified analysis of the population. Third, since all the participants included in our study were restricted to European database, our findings may not be generalizable to other ethnic groups, and therefore the analysis of larger and more diverse cohorts is likely to provide additional insight. Finally, immunophenotypes may play an important role in HIV-1 survival or disease progression^10,80,81^. However, our MR analysis did not solve this association, so further research should analyze whether immunophenotypes play a role in the invasion of HIV-1 and even the prognosis of AIDS.

## Conclusion

In conclusion, we have delineated that 26 immunophenotypes might have a causal influence on HIV-1 acquisition through a comprehensive bidirectional MR analysis. 39 SMR-identified genes revealed critical pathways linking immunophenotypes to HIV-1 susceptibility. It might provide a new path for researchers to explore the biological mechanisms of HIV-1 acquisition and can lead to exploration of earlier intervention and treatment.

## Supplementary Information

Supplementary Information 1.

Supplementary Information 2.

Supplementary Information 3.

Supplementary Information 4.

Supplementary Information 5.

## Data Availability

Data sharing statement Publicly available datasets were analyzed in this study. This data can be found here: (https://kcl.figshare.com/articles/dataset/GWAS_Summary_statistics_-_HIV-1_acquisition_meta-analysis_-_Duarte_et_al_2022/18166406/1).
