# Supplementary material for: Causal role of immunophenotypes in HIV-1 acquisition: insights from Mendelian randomization analysis
Source: Sci Rep. 2025 Jul 2;15:23618. doi: 10.1038/s41598-025-07962-y (PMC12223304; doi:10.1038/s41598-025-07962-y)
Supplement: Supplementary file 1 — Supplementary Information 1. [file 41598_2025_7962_MOESM1_ESM.pdf]

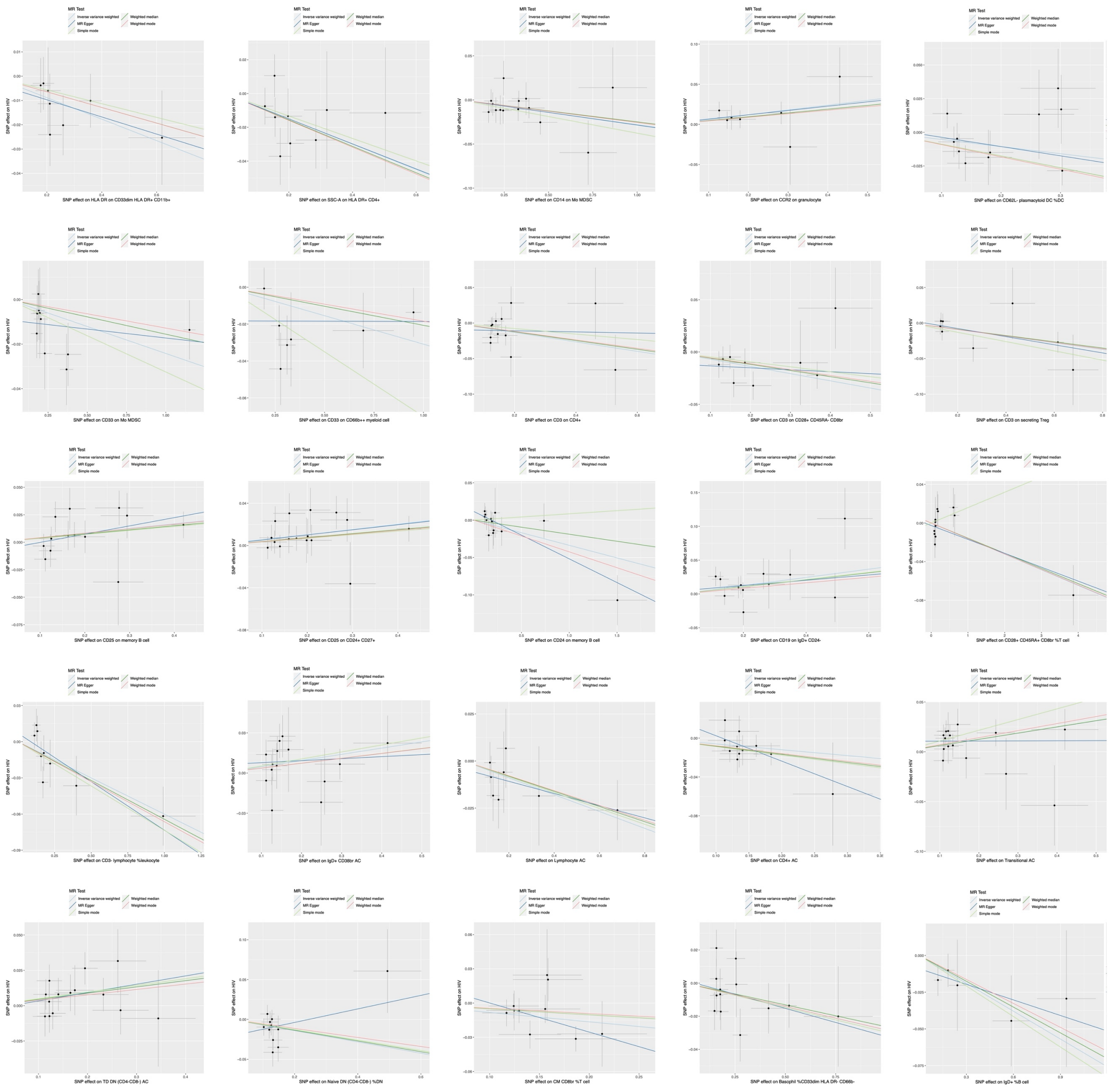

Supplementary Figure 1. Scatterplot of estimated causal effects of immunophenotypes on HIV

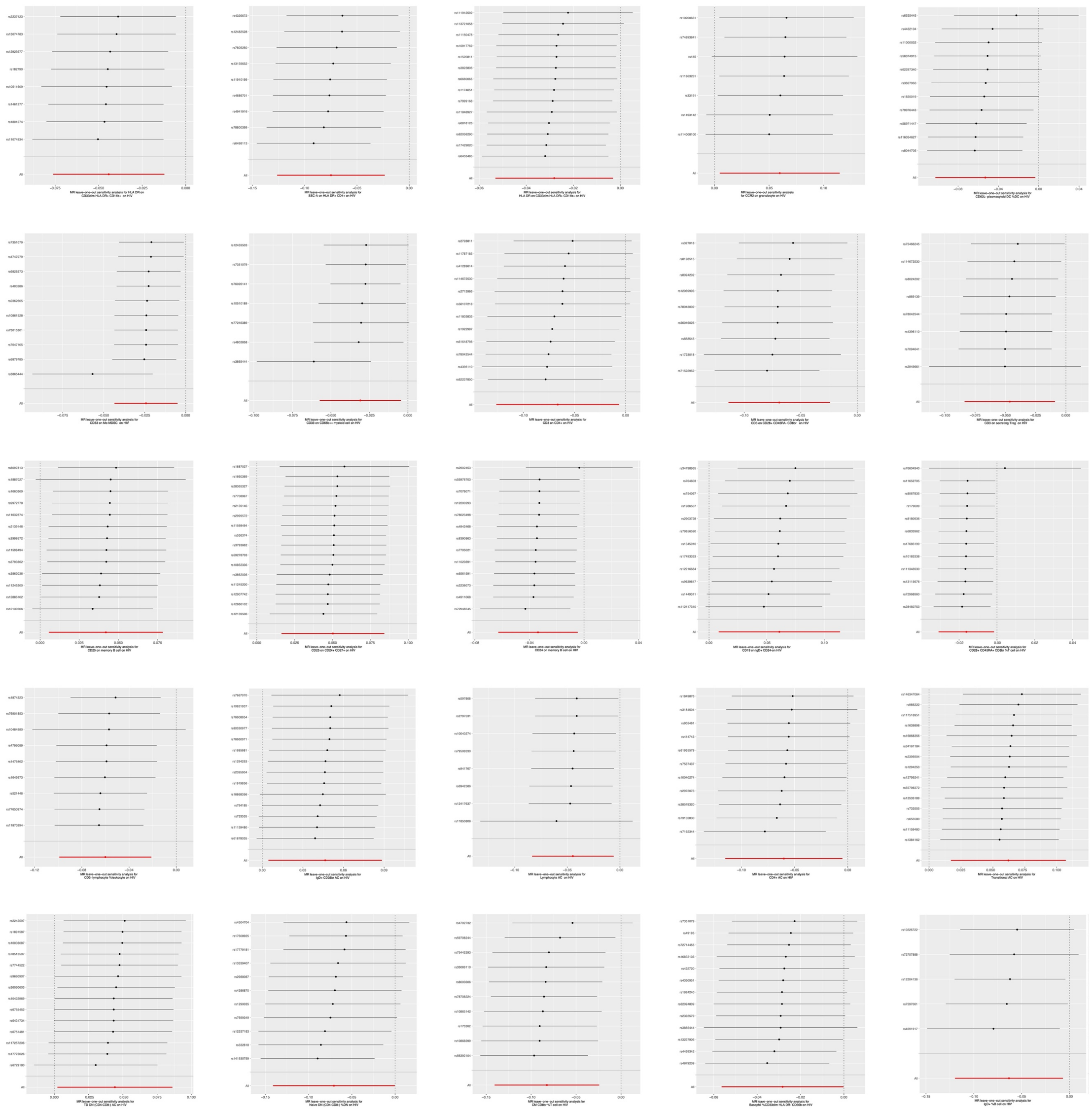

Supplementary Figure 2. Leave-one-out plots of immunophenotypes on HIV.

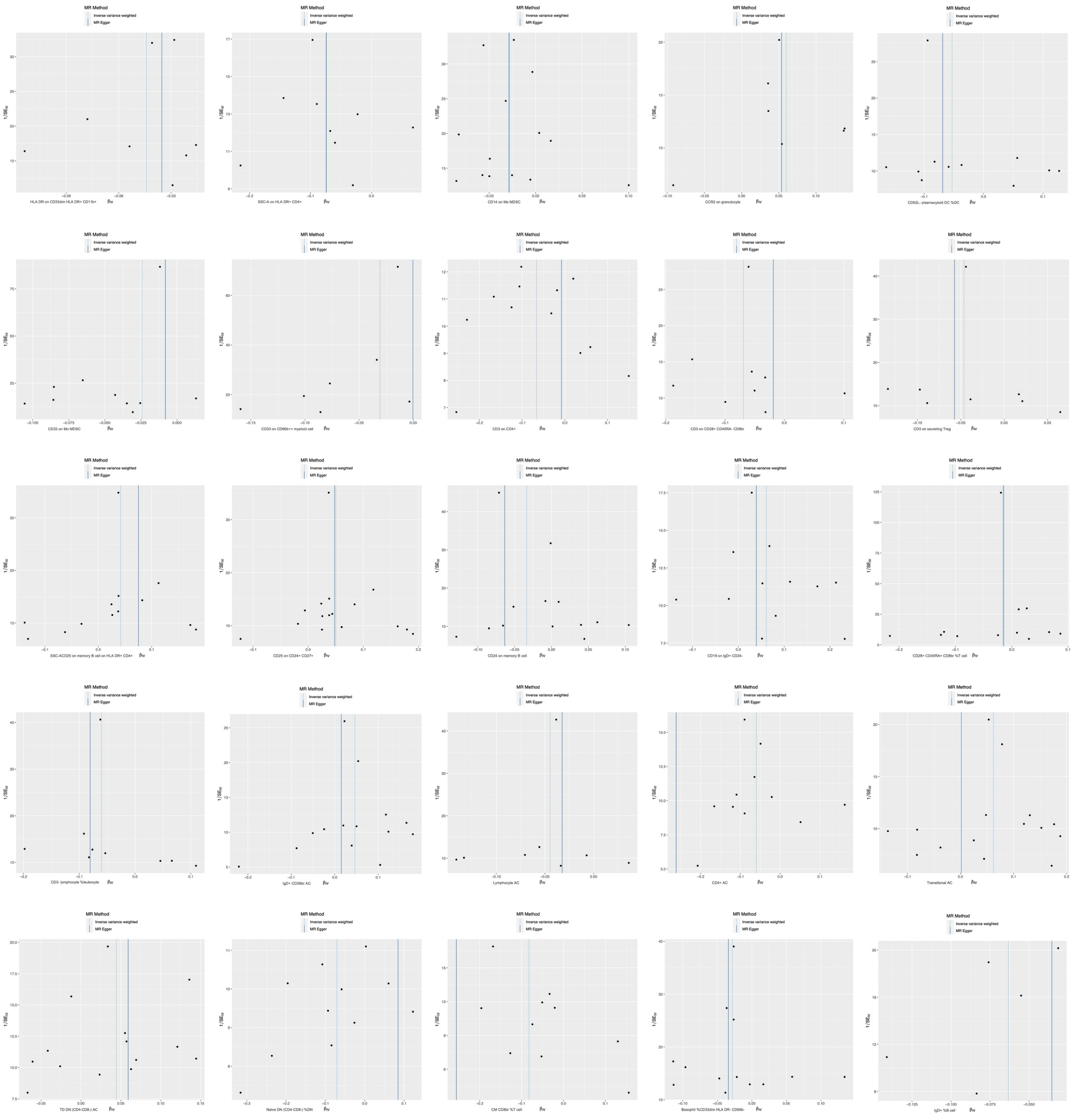

Supplementary Figure 3. Funnel plot of immunophenotypes on HIV.

Note: We found that for GCST90001640 (Relative count, TBNK, CD3- lymphocyte % lymphocyte), the limited number of instrumental SNPs (n=2) precluded the generation of scatter plots, LOO, and funnel plots. Consequently, our supplementary materials include visualizations for 25 immunophenotypes.

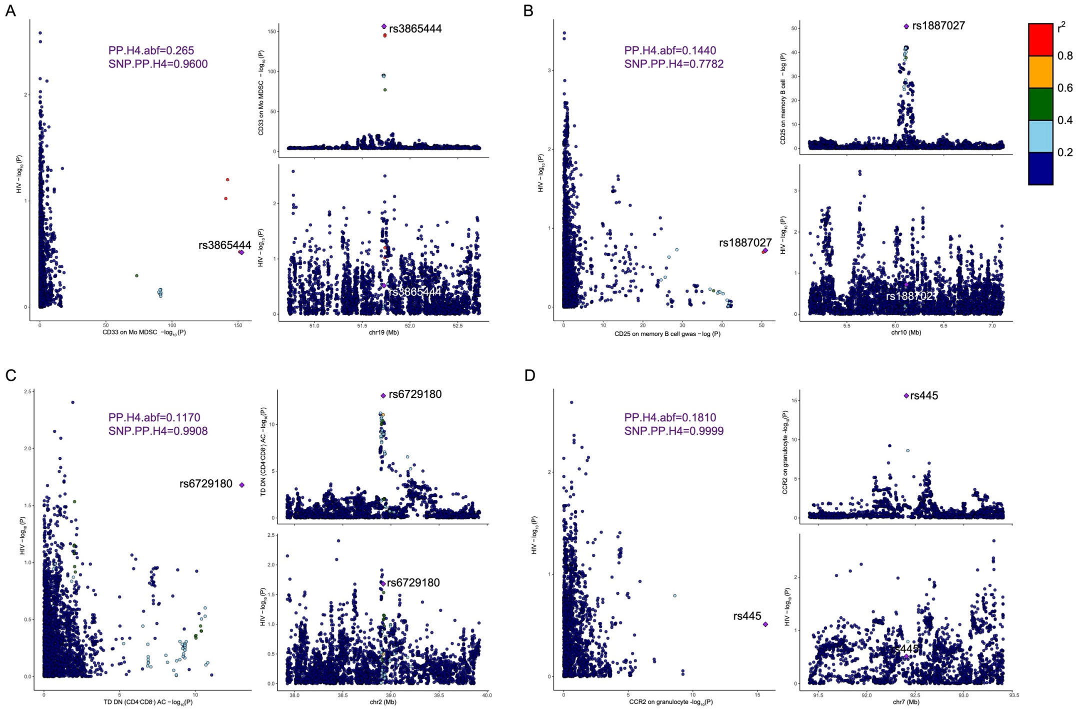

Supplementary Figure 4. Colocalization of four immunophenotypes and HIV. (A) CD33 on Mo MDSC, (B) CD25 on memory B cell, (C) TD DN (CD4- CD8-) and AC, (D) CCR2 on granulocyte with HIV. Each dot represented a genetic variant with the candidate causal variant. Top SNP was shown as a purple diamond.
